# *Eg5* as a Prognostic Biomarker and Potential Therapeutic Target for Hepatocellular Carcinoma

**DOI:** 10.3390/cells10071698

**Published:** 2021-07-05

**Authors:** Yu-Yun Shao, Nai-Yun Sun, Yung-Ming Jeng, Yao-Ming Wu, Chiun Hsu, Chih-Hung Hsu, Hey-Chi Hsu, Ann-Lii Cheng, Zhong-Zhe Lin

**Affiliations:** 1 Graduate Institute of Oncology, National Taiwan University College of Medicine, 1, Sec. 1, Ren’ai Rd., Taipei 100, Taiwan; yuyunshao@gmail.com (Y.-Y.S.); naiyunsun@hotmail.com (N.-Y.S.); hsuchiun@gmail.com (C.H.); chihhunghsu@ntu.edu.tw (C.-H.H.); alcheng@ntu.edu.tw (A.-L.C.); 2 Department of Oncology, National Taiwan University Hospital, 7, Chung-Shan S. Rd., Taipei 100, Taiwan; 3 Department of Pathology, National Taiwan University Hospital, 7, Chung-Shan S. Rd., Taipei 100, Taiwan; mrna0912@gmail.com (Y.-M.J.); heychi@ntu.edu.tw (H.-C.H.); 4 Graduate Institute of Pathology, National Taiwan University College of Medicine, 1, Sec. 1, Ren’ai Rd., Taipei 100, Taiwan; 5 Department of Surgery, National Taiwan University Hospital, 7, Chung-Shan S. Rd., Taipei 100, Taiwan; wyaoming@ntuh.gov.tw; 6 Department of Medical Oncology, National Taiwan University Cancer Center, 57, Lane 155, Sec. 3, Keelung Rd., Taipei 106, Taiwan; 7 Department of Internal Medicine, National Taiwan University Hospital, 7, Chung-Shan S. Rd., Taipei 100, Taiwan

**Keywords:** *Eg5*, hepatocellular carcinoma, kinesin, mitosis, prognosis

## Abstract

Background: The kinesin *Eg5*, a mitosis-associated protein, is overexpressed in many cancers. Here we explored the clinical significance of *Eg5* in hepatocellular carcinoma (HCC). Methods: HCC tissues from surgical resection were collected. Total RNA was prepared from tumorous and nontumorous parts. *Eg5* expression levels were correlated with overall survival (OS) and disease-free survival (DFS). In vitro efficacy of LGI-147, a specific *Eg5* inhibitor, was tested in HCC cell lines. In vivo efficacy of *Eg5* inhibition was investigated in a xenograft model. Results: A total of 108 HCC samples were included. The patients were divided into three tertile groups with high, medium, and low *Eg5* expression levels. OS of patients with low *Eg5* expression was better than that of patients with medium and high *Eg5* expression (median, 155.6 vs. 75.3 vs. 57.7 months, *p* = 0.002). DFS of patients with low *Eg5* expression was also better than that of patients with medium and high *Eg5* expression (median, 126.3 vs. 46.2 vs. 39.4 months, *p* = 0.001). In multivariate analyses, the associations between *Eg5* expression and OS (*p* < 0.001) or DFS remained (*p* < 0.001). LGI-147 reduced cell growth via cell cycle arrest and apoptosis and induced accumulation of abnormal mitotic cells. In the xenograft model, the tumor growth rate under LGI-147 treatment was significantly slower than under the control. Conclusion: High *Eg5* expression was associated with poor HCC prognosis. In vitro and in vivo evidence suggests that *Eg5* may be a reasonable therapeutic target for HCC.

## 1. Introduction

Treatment for unresectable hepatocellular carcinoma (HCC) remains challenging despite improvements in treatment modalities including antiangiogenic targeted therapy and immune checkpoint inhibitors [1]. For diseases refractory or unamenable to transarterial chemoembolization, combined targeted therapy and immunotherapy can produce an objective response rate of approximately 30% [2,3,4,5], which leaves much room for improvement. Moreover, patients who fail first-line systemic therapy exhibit poor prognosis [6]. Therefore, novel modalities of systemic therapies for HCC are urgently required.

We and other investigators have discovered that mitosis regulators, such as Aurora kinases A and B, are frequently overexpressed in HCC cells and have been associated with poor HCC prognosis [7,8,9]. Kinesins are a superfamily of motor proteins that participate in the organelle transport and mitosis [10,11]. Overexpression of *Eg5*, a kinesin, may lead to genomic instability and tumor formation in mice [12]. High *Eg5* expression in tumor tissues is also associated with poor prognosis in breast and laryngeal cancers [13,14].

In addition, mitosis regulators also can serve as potential cancer treatment therapeutic targets. Taxanes and vinka alkaloids, chemotherapeutic agents effective against multiple cancers, interfere with microtubules and hence mitotic function [15]. Numerous studies have indicated the potential of kinesin inhibitors as treatment for various cancers [10,16]. Among them, *Eg5* inhibitors have been reported to be effective in preclinical models of melanoma as well as breast, ovarian, and prostate cancers [17,18,19,20].

The role of *Eg5* in prognosis prediction and as a therapeutic target related to HCC is unclear. We examined the association between *Eg5* expression in surgically resected tumor tissues and HCC prognosis. We also tested the in vitro and in vivo efficacy of *Eg5* inhibition against HCC.

## 2. Methods

### 2.1. Patient Samples

We assessed unifocal primary HCC tissues from 108 patients who underwent surgical total tumor resection between January 1987 and January 2008. Comprehensive pathologic assessments and regular clinical follow-ups were performed at the National Taiwan University Hospital (NTUH), as described previously [7]. Patients with evidence of regional lymph node or distant metastasis were excluded. This study was approved by the Research Ethics Committee of NTUH.

### 2.2. Quantitative Reverse Transcription-Polymerase Chain Reaction

The extraction of total RNA and complementary DNA of paired HCCs and nontumorous liver tissues was prepared as described previously [21]. Gene expression assays for *Eg5* were performed using quantitative reverse transcription polymerase chain reaction with the TaqMan^®^ Gene Expression Master Mix and an *Eg5* probe (Hs00189698_m1), with *GAPDH* as a control (Hs99999905_m1; Applied Biosystems, Foster City, CA, USA). The expression levels of *Eg5* and *GAPDH* were determined through 45 thermal cycles of 30 s at 95 °C and 60 s at 60 °C. All experiments were performed in duplicate. Quantitative data were expressed as the numbers of cycles required to reach a specific threshold of detection (C_T_ value) during the exponential amplification phase. The relative quantification of *Eg5* expression was calculated using the comparative threshold cycle (2^−∆∆CT^) method (∆C_T_ = C_T (*Eg5*)_ − C_T (GAPDH)_, ∆∆C_T_ = ∆C_T_ (tumor tissue) − ∆C_T_ (normal liver tissue)) [21].

### 2.3. Cell Culture and Reagents

The liver cancer cell lines HepG2, Hep3B, and PLC5 were maintained in Dulbecco’s modified Eagle’s medium plus 10% fetal bovine serum, supplemented with 100 U/mL penicillin and 100 μg/mL streptomycin. Cells were cultured in a humidified incubator with 5% CO_2_ at an air temperature of 37 °C.

LGI-147 was provided by Novartis Pharma AG (Basel, Switzerland). The biochemical half maximal inhibitory concentration (IC_50_) of LGI-147 for *Eg5* is 0.6 nM (unpublished data provided by Novartis Pharma AG).

### 2.4. Cell Viability

A total of 5 × 10^4^ liver cancer cells were seeded in 6-well plates. After overnight culture, cells were treated with dimethyl sulfoxide or LGI-147 for 72 h. Cell viability was quantified using the trypan blue exclusion assay as described previously [7].

### 2.5. Cell-Free Kinesin ATPase End-Point Assay

Purified kinesin motor proteins, namely *Eg5*, centromere-associated protein E (CENP-E), mitotic kinesin-like protein-1 (MKLP-1), and BimC, were purchased from Cytoskeleton, Inc. (Denver, CO, USA). We used the HTS Kinesin ATPase End-Point Biochem kit (Cytoskeleton, Inc.) to examine kinesin activity [22]. Inhibition of kinesin activity was calculated using the following formula: average % = ((average untreated − average treated)/average untreated) × 100.

### 2.6. Immunofluorescence Staining

Morphologic changes in the mitotic spindles, centromeres, and chromosomes of the liver cancer cells were detected through immunofluorescence staining, which was performed as previously described [8]. Primary antibodies against α-tubulin (1:100, Sigma-Aldrich) or γ-tubulin (1:100, Sigma-Aldrich) were used. Cells were then incubated with fluorescein-conjugated secondary antibodies (1:200; Santa Cruz Biotechnology, Inc.) for 1 h. Nuclei were counterstained with 0.5 μg/mL 4′,6-diamidino-2-phenylindole (DAPI) for 15 min. Images were captured using a confocal microscope (Leica TCS SP2, Wetzlar, Germany).

### 2.7. Cell Cycle and Apoptosis Analyses

Cells in logarithmic growth were incubated with either LGI-147 or dimethyl sulfoxide for 24 to 72 h. Cells were trypsinized and fixed in 70% methanol overnight and labeled with 0.5 to 1 mL propidium iodide at 50 μg/mL. Cell cycle profiles were determined using a FACSCaliber (Becton Dickinson, San Jose, CA, USA).

The sub-G1 assay by flow cytometry was used to determine apoptotic cell numbers. Western blotting was performed according to standard protocols using an anti-cleaved poly(ADP-ribose) polymerase (PARP) antibody (Cell Signaling Technology, Beverly, MA, USA) and an anti-β-actin antibody (Sigma-Aldrich) to detect apoptotic signals.

### 2.8. Xenograft Animal Studies

Animal studies were conducted according to the guidelines of the Institutional Animal Care and Use Committee of NTUH. LGI-147 was prepared in 20% Captisol (Captisol, San Diego, CA, USA) solution. All experiments were performed on 5-week-old male BALB/c nude mice purchased from BioLASCO, Ltd. (Taipei, Taiwan). PLC5 cells were injected subcutaneously into the right flanks (2 × 10^6^/flank in 200 μL) of the mice. When tumor volume reached approximately 200 mm^3^, the mice were treated with intravenous injection of LGI-147 or a vehicle twice a week. Tumor size was estimated twice a week, and the body weight was monitored daily.

### 2.9. Statistical Analysis

All statistical analyses were performed using SAS software version 9.4 (SAS Institute Inc., Cary, NC, USA). A two-sided *p* value of ≤0.05 was considered statistically significant. For continuous variables such as tumor size and *Eg5* expression, either the independent *t* test or one-way analysis of variance was used for between-group comparisons. The Pearson correlation coefficient was calculated to examine the correlation between age and *Eg5* expression. The Kaplan–Meier method was used to estimate survival outcomes. To compare survival between groups, the log-rank test and a Cox proportional hazards model were used in univariate and multivariate analysis, respectively. Overall survival (OS) was defined as the period from the surgery date until the date of death. Disease-free survival (DFS) denoted the period from the surgery date until tumor recurrence or the date of death, whichever occurred first. Minimal follow-up duration was 5 years. At the end of the follow-up session in August 2019, only 17 patients were still alive.

## 3. Results

### 3.1. Eg5 Expression and HCC Prognosis

A total of 108 unifocal primary HCC samples from patients who received curative surgery for HCC were included. The mean patient age was 54.7 years, and 19% were female (Table 1). Hepatitis B virus and hepatitis C virus infection was detected in 69% and 29% of patients, respectively. The mean RNA expression of *Eg5* in tumor tissues compared with that in nontumor tissues was 8.3. *Eg5* expression was not significantly associated with patient demographic characteristics, tumor extent, or tumor grade (Table 1).

**Figure 1:**
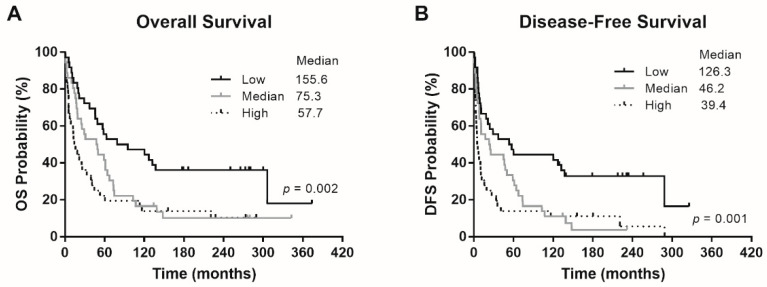
Survival outcomes of the patients. (**A**) Overall survival (OS) and (**B**) disease-free survival (DFS) according to patients’ *Eg5* expression levels. *p* values were conducted using the log-rank test.

The patients were divided into three tertile groups with high (>5.33), medium (1.4–5.33), and low *Eg5* (<1.4) mRNA expression levels. The 24-month OS rates of patients with low, medium, and high *Eg5* expression were 75%, 63.9%, and 41.7%, respectively. The median OS of patients with low, medium, and high *Eg5* expression was 155.6, 75.3, and 57.7 months, respectively (Figure 1A). The 24-month DFS rates of patients with low, medium, and high *Eg5* expression were 58.3%, 50.0%, and 25.0%, respectively. The median DFS of patients with low, medium, and high *Eg5* expression was 126.3, 46.2, and 39.4 months, respectively (Figure 1B). Thus, the patients with low *Eg5* expression exhibited the best survival outcomes, as compared with two groups of patients with high and medium *Eg5* expression, in OS (*p* = 0.002) and DFS (*p* = 0.001). In other words, high *Eg5* expression seems to correlate with tumor progression and hence poor patient survival.

After adjustment for other clinicopathological variables, including gender, age, tumor stage, hepatitis etiology, and α-fetoprotein level, low *Eg5* expression remained an independent predictor of better OS (hazard ratio (HR) 0.377, *p* < 0.001) and DFS (HR 0.334, *p* < 0.001; Table 2).

### 3.2. Eg5 Inhibition Reduced HCC Cell Viability

**Figure 2:**
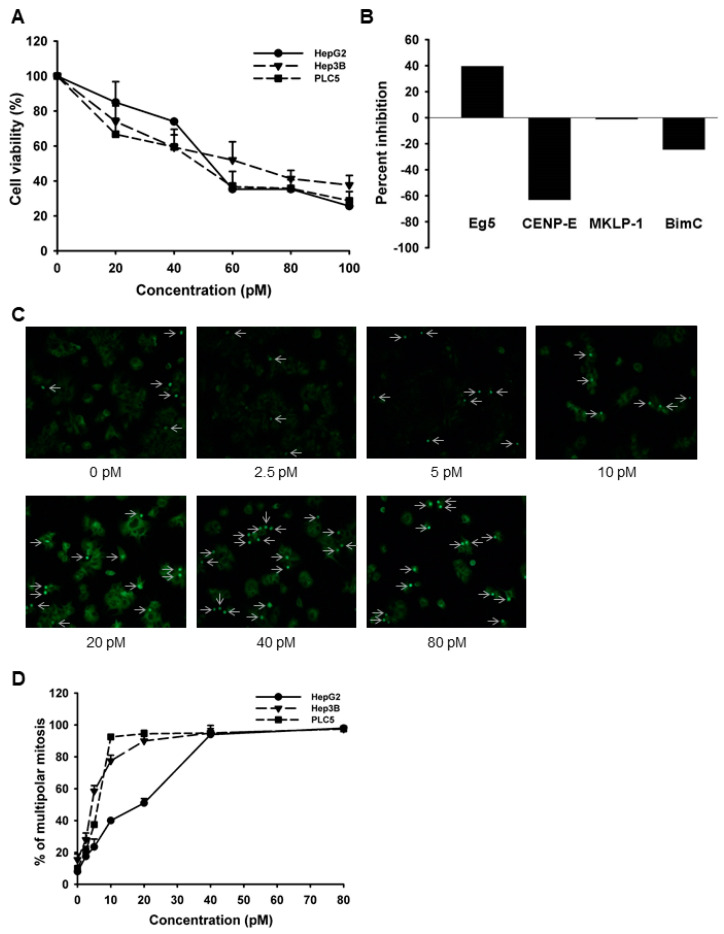
(**A**) Cell viability analysis. Cells were treated with LGI-147 at the indicated concentrations for 72 h, and their viability was calculated using the trypan blue exclusion assay. (**B**) Activity of kinesin motor proteins. Purified *Eg5*, CENP-E, MKLP-1, and BimC underwent a cell-free kinesin ATPase end-point assay either under 50 pM of LGI-147 or not. Values shown are percentages of inhibition corrected by the controls. (**C**,**D**) Accumulation of abnormal mitotic cells (indicated by the arrows) due to LGI-147 treatment. After 24 h of treatment with the indicated concentrations of LGI-147, the HCC cells were fixed and stained with an anti-α-tubulin antibody (green). (**C**) Images shown are experiments on PLC5 cells captured through a fluorescence microscope. (**D**) Quantified results of Figure 2C.

Because our results suggested that high *Eg5* expression seems to correlate with tumor progression and hence poor patient survival, we then tried to test the effect of *Eg5* inhibition on HCC cells. We first performed the trypan blue exclusion assay to test the antiproliferative effects of LGI-147, an *Eg5* inhibitor, on multiple HCC cell lines, including HepG2, Hep3B, and PLC5 cells. LGI-147 reduced cell viability in all cell lines in a dose-dependent manner (Figure 2A). The IC_50_ at 72 h for the HepG2, Hep3B, and PLC5 cells were 53.59, 59.6, and 43.47 pM, respectively. We examined the specific kinase inhibitory activity of LGI-147 using the cell-free kinesin ATPase assay. LGI-147 inhibited the activity of *Eg5* but not that of other kinesins such as CENP-E, MKLP-1, and BimC (Figure 2B).

### 3.3. Cellular Effects of Eg5 Inhibition in HCC Cells

**Figure 3:**
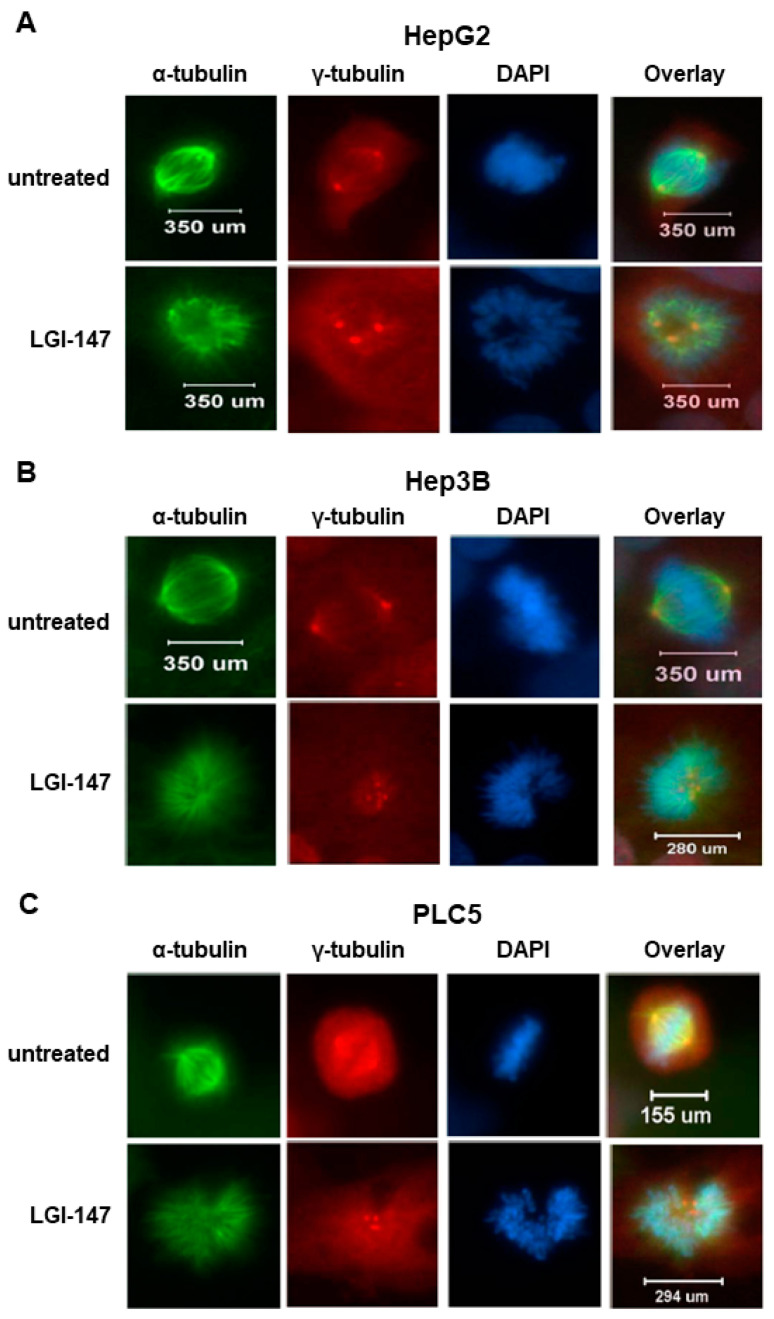
(**A**–**C**) Abnormal monopolar spindle formation in HCC cells under LGI-147 treatment during prometaphase. After 24 h treatment with the vehicle or 50 pM of LGI-147, the HCC cells were fixed and stained with anti-α-tubulin (green) and anti-γ-tubulin antibodies (red) and DAPI (blue). The images were captured using a confocal microscope (63× objective).

To analyze the mitotic interference of *Eg5* inhibition, we examined the morphological changes in mitotic spindles and chromosomes in HCC cells treated with LGI-147. The accumulation of abnormal mitotic cells induced by LGI-147 was dose dependent. After treatment with 40 pM of LGI-147, more than 75% of HCC cells showed abnormal mitotic features (Figure 2C,D). LGI-147 induced an accumulation of prometaphase cells with disturbed centrosome maturation and abnormal monopolar spindles (Figure 3A–C).

**Figure 4:**
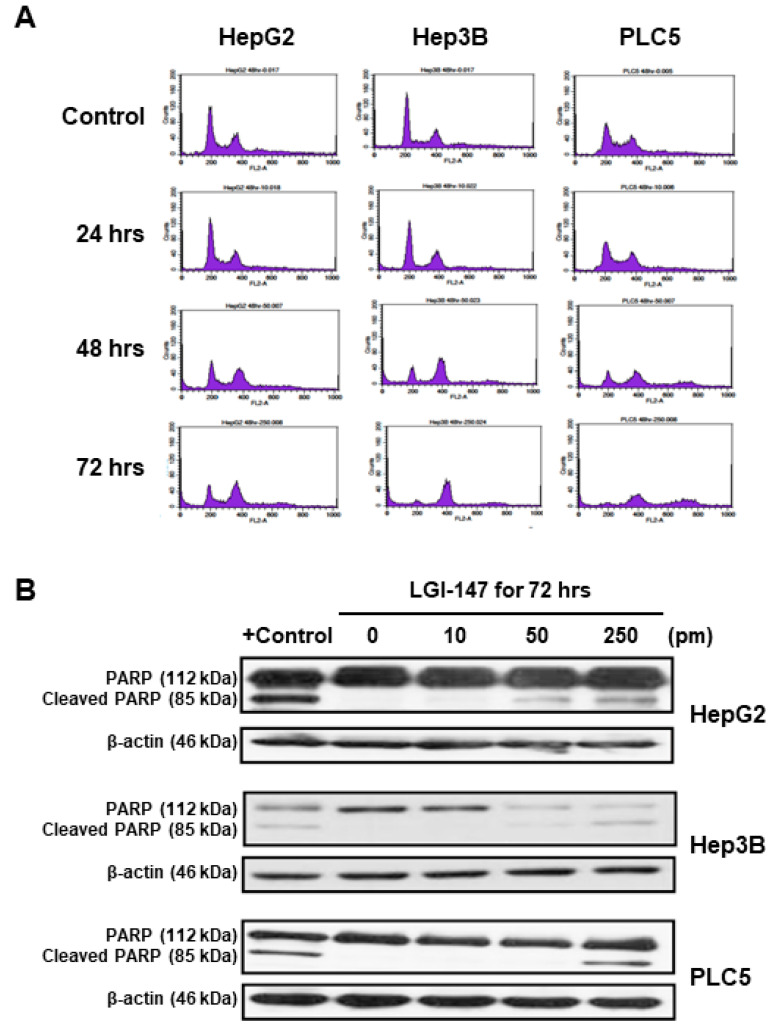
(**A**) Cell cycle disturbance in the HCC cells. Cells were treated with 50 pM LGI-147 for 24 to 72 h, and then stained with propidium iodide. DNA content was analyzed using the flow cytometry. Data shown are representative of three independent experiments. (**B**) Cell apoptosis. The HCC cells were treated with a vehicle, positive control (1 μM doxorubicin), or indicated concentrations of LGI-147 for 72 h. PARP cleavage was detected using Western blotting.

Because mitotic interference may induce cell cycle disturbance and cell death, we investigated the effects of LGI-147 on HCC cell cycle progression and apoptosis. As shown in Figure 4A, *Eg5* inhibition by LGI-147 treatment resulted in time-dependent cell cycle arrest and accumulation of tetraploid cells. LGI-147 treatment also led to the appearance of octoploid cells, which preceded cell death, particularly in the PLC5 cell line.

**Figure 5:**
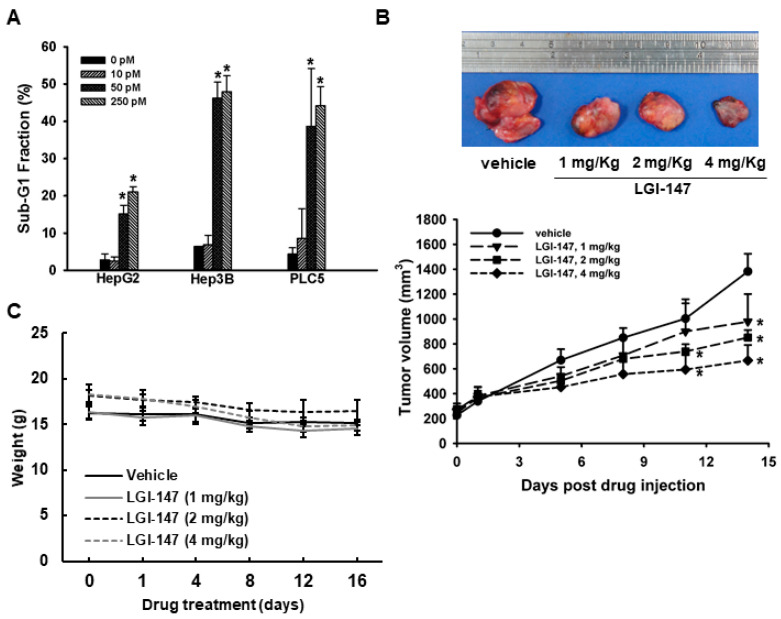
(**A**) Apoptosis due to *Eg5* inhibition. Sub-G1 DNA content in HCC cells treated with vehicle or LGI-147 at the indicated concentrations for 72 h. The percentage of cells with sub-G1 DNA content is shown. Columns denote means, and bars denote standard deviations (n = 3). *, *p* < 0.05. (**B**,**C**) Suppression of in vivo tumor growth by LGI-147. PLC5 cells were injected subcutaneously into 5-week-old BALC/c mice (n = 6 per group). When tumor volume reached approximately 200 mm^3^, the mice were treated with intravenous injections of LGI-147 twice a week at the indicated doses. (**B**) Tumor size was estimated twice a week, and (**C**) body weight was monitored daily.

*Eg5* inhibition by LGI-147 treatment also induced dose-dependent apoptosis. After 72 h LGI-147 treatment, PARP cleavage was detected (Figure 4B), and the sub-G1 fractions of the HCC cells increased significantly (*p* < 0.05, Figure 5A), particularly under an LGI-147 concentration of ≥50 pM.

### 3.4. Eg5 Inhibition Reduced In Vivo HCC Tumor Growth

To determine the in vivo antitumor efficacy of LGI-147, a PLC5 xenograft model was established. PLC5 tumor growth was significantly suppressed by LGI-147 treatment (Figure 5B). At day 14 of LGI-147 treatment, the mean tumor volumes of mice treated at 2 mg/Kg (851.97 mm^3^) and 4 mg/kg (666.94 mm^3^) were significantly lower than that of the control group (1382.21 mm^3^; *p* < 0.05 for both). Mouse weight did not differ significantly between groups (Figure 5C).

## 4. Discussion

In this study, we observed an association between high tumor expression of *Eg5* and poor HCC prognosis. The 24-month OS rates of patients with low and high *Eg5* expression of 75% and 41.7% differed substantially. Even after adjustment for other clinicopathological variables, *Eg5* expression remained an independent predictor of OS and DFS. The preclinical HCC models demonstrated the therapeutic potential of *Eg5* inhibition through a novel *Eg5* inhibitor, LGI-147. *Eg5* inhibition by LGI-147 interfered with mitosis, halted the cell cycle, and induced apoptosis in the HCC cells. The HCC xenograft model also demonstrated the in vivo antitumor efficacy of LGI-147.

Inhibition of cell proliferation through mitosis is a clinically effective anticancer intervention [23]. As our previous studies have demonstrated, the overexpression of Aurora kinases A and B, essential mitotic kinases, in HCC cells is associated with poor HCC prognosis [7,9]. Furthermore, Aurora kinase inhibitors have potent anticancer effects in human HCC [7,9]. Elucidation of the prognostic significance of *Eg5* expression and the antitumor efficacy of specific *Eg5* inhibitors is essential to establish *Eg5* as a therapeutic target for HCC. Therefore, the findings of the present study provide a rationale for the clinical development of specific *Eg5* inhibitors for HCC treatment. Our findings regarding the prognostic value of *Eg5* expression are generally consistent with those of a previous study [24], although that study did not analyze DFS.

The past decade has seen the identification of multiple anticancer small-molecule inhibitors targeting mitotic machinery, including Aurora kinases, Polo-like kinase 1, *Eg5*, and CENP-E. Their cellular consequences are typically disturbance of the cell cycle, suppression of cell proliferation, and induction of apoptosis at mitotic phase or following mitotic slippage [25]. *Eg5* is a promising anticancer therapeutic target because, as with other kinesins such as CENP-E, it is critically involved in centrosome maturation, spindle assembly, chromosome segregation, and cytokinesis [16]. In the current study, we used LGI-147, a specific *Eg5* inhibitor that did not inhibit the activity of other kinesins such as CENP-E, MKLP-1, and BimC. The IC_50_ of LGI-147 on cell viability at the pM level was extremely low. The therapeutic potential of other *Eg5* inhibitors such as AZD4877 [26,27] and filanesib [28,29,30] has been demonstrated in several phase I or II clinical trials for cancers other than HCC. Our findings may provide a basis for the development of LGI-147 or other *Eg5* inhibitors as HCC therapeutics.

Our study had some limitations. First, we only used one method of *Eg5* inhibition because we did not have access to *Eg5* inhibitors other than LGI-147. However, as mentioned, LGI-147 had high specificity; therefore, the possibility of an off-target effect of LGI-147 as the primary mechanism is low. Second, we did not examine the peripheral blood cell counts of mice under LGI-147 treatment. Because mitosis inhibitors may affect all dividing cells, bone marrow suppression can be primary toxicity. However, such problems can be addressed in phase 1 clinical trials or resolved through scheduling. In in vivo studies, tumor size reduction may result from mechanisms other than apoptosis, such as tissue inflammatory or stromal changes. These should be explored in further research.

In conclusion, high *Eg5* expression was associated with poor HCC prognosis. *Eg5* inhibition with LGI-147 demonstrated promising in vitro and in vivo efficacy against HCC cells, suggesting that *Eg5* is a potential clinical prognostic factor and therapeutic target for HCC.

## Figures and Tables

**Table 1:** Patient characteristics and their associations with *Eg5* expression.

Variables	N (%)	*Eg5* ^†^	
		Mean ± SD	*p*
Total	108 (100)	8.3 ± 16.0 ^#^	
Mean age (SD, years)	54.7 (13.4) ^#^		
Gender			0.887
Female	21 (19)	8.7 ± 10.6	
Male	87 (81)	8.2 ± 17.1	
Hepatitis virus			
HBsAg positive	75 (69)	8.3 ± 15.6	0.964
Anti-HCV positive	31 (29)	6.9 ± 16.6	0.578
AJCC stage			0.573
I	46 (43)	7.5 ± 19.8	
II	32 (30)	7.0 ± 8.5	
III	30 (28)	10.9 ± 15.8	
Tumor size			0.835
>5 cm	48 (44)	8.7 ± 19.3	
≤5 cm	60 (56)	8.0 ± 12.9	
Tumor grade			0.683
1	26 (24)	10.3 ± 24.4	
2	51 (47)	7.0 ± 14.0	
3	31 (29)	8.8 ± 9.1	
AFP > 400 ng/mL	40 (37)	9.4 ± 14.8	0.573
Child-Pugh status			0.828
A	100 (93)	8.4 ± 16.4	
B	8 (7)	7.1 ± 9.7	

Abbreviations: SD = standard deviation; HBsAg = hepatitis B virus surface antigen; HCV = hepatitis C virus; AJCC = American Joint Committee on Cancer; AFP = α-fetoprotein. ^#^ Age had a weakly positive correlation with *Eg5* expression (r = 0.013, *p* = 0.897). **^†^** Calculated using the method described in Section 2.2. *p* values were conducted using the independent t test or one-way analysis of variance.

**Table 2:** Multivariate analysis of potential predictors of overall survival and disease-free survival using Cox proportional hazards models.

Variables	Overall Survival			Disease-Free Survival		
	*p*	HR	95% CI	*p*	HR	95% CI
*Eg5* low (vs. high)	<0.001	0.377	0.214–0.665	<0.001	0.334	0.187–0.596
*Eg5* medium (vs. high)	0.391	0.793	0.468–1.346	0.352	0.773	0.449–1.330
Age	0.133	1.014	0.996–1.033	0.594	1.005	0.988–1.022
Male (vs. female)	0.924	0.971	0.537–1.759	0.286	0.732	0.412–1.299
HBsAg positive	0.007	2.580	1.302–5.112	0.065	1.880	0.961–3.675
Anti-HCV positive	0.063	1.761	0.969–3.201	0.188	1.498	0.821–2.733
AJCC stage I (vs. III)	<0.001	0.314	0.184–0.538	0.004	0.464	0.274–0.784
AJCC stage II (vs. III)	<0.001	0.305	0.168–0.552	<0.001	0.372	0.209–0.663
AFP > 400 ng/mL	0.486	1.185	0.735–1.911	0.056	1.599	0.988–2.589
Child B (vs. A)	0.355	1.458	0.656–3.237	0.306	1.531	0.677–3.460

Abbreviations: OS = overall survival; DFS = disease-free survival; HR = hazard ratio; CI = confidence interval; HBsAg = hepatitis B virus surface antigen; HCV = hepatitis C virus; AJCC = American Joint Committee on Cancer; AFP = α-fetoprotein.

## Data Availability

All data of this study were included in this manuscript.
